# Circulating MicroRNAs in Cancer: Potential and Challenge

**DOI:** 10.3389/fgene.2019.00626

**Published:** 2019-07-18

**Authors:** Mengying Cui, Hongdan Wang, Xiaoxiao Yao, Dan Zhang, Yingjun Xie, Ranji Cui, Xuewen Zhang

**Affiliations:** ^1^Department of Hepatobiliary and Pancreatic Surgery, The Second Hospital of Jilin University, Changchun, China; ^2^Department of Anesthesiology, The First Hospital of Jilin University, Changchun, China; ^3^Jilin Provincial Key Laboratory on Molecular and Chemical Genetic, The Second Hospital of Jilin University, Changchun, China

**Keywords:** miRNAs, circulating, cancer, biomarker, communication, therapy, challenge

## Abstract

MicroRNAs (miRNAs) are endogenous non-coding small RNA molecules that can be secreted into the circulation and exist in remarkably stable forms. Like intercellular miRNAs, circulating miRNAs participate in numerous regulations of biological process and expressed aberrantly under abnormal or pathological status. The quality and quantity changes of circulating miRNAs are associated with the initiation and progression of cancer and can be easily detected by basic molecular biology techniques. Consequently, considerable effort has been devoted to identify suitable extracellular miRNAs for noninvasive biomarkers in cancer. However, several challenges need to be overcome before the practical application. In this review, we discuss several issues of circulating miRNAs: biological function and basic transport carriers; extracellular cell communication process; roles as reliable cancer biomarkers and usage in targeted cancer therapy; and challenges for clinical application.

## Introduction

MicroRNA (miRNA) was first discovered as the product of the *lin-4* gene in *Caenorhabditis elegans* in 1993 (Lee et al., 1993; Wightman et al., 1993). The small non-coding RNAs (19-22nt) develop post-transcriptional regulation by mRNA cleavage or translation repression, which depended on the complementarity degree of miRNA-mRNA. mRNA cleavage occurs when there is a perfect match, whereas imperfect combination results in gene repression (Bartel, 2004). A large number of studies have confirmed the role of microRNAs in various cancer-associated biological processes, such as proliferation, differentiation, apoptosis, metabolism, invasion, metastasis, and drug resistance. The pathological origin of cancer has also been proven to be directly related to the dysregulation of miRNAs. Moreover, miRNAs are tissue-specific. Different tumors have distinctive miRNA expression profiles. So far, the basic biogenesis and function of the intracellular miRNAs have been reviewed in a number of contexts. On the other hand, the presence of extracellular RNAs in serum/plasma was described first by Bartel (Bartel, 2004; Kibel, 2009) and various miRNAs are proved to exist in a stable cell-free form in body fluids and other extracellular environments, including plasma, serum, urine, saliva, seminal, ascites, amniotic pleural effusions, and cerebrospinal fluid (Weber et al., 2010; Cortez et al., 2011; Husted et al., 2011; Alečković and Kang, 2015; Kibel, 2009). Studies suggested that they are injected to the circulation in different ways. Parts are due to the passive leakage of apoptosis, necrosis, or the environment of inflammation, and parts are secreted actively by exosomes/microvesicles, lipoproteins, and RNA-protein complex (Arroyo et al., 2011; Cheng, 2015). Furthermore, specific miRNAs are selected to pack into exosomes and become one of the most important aspects of the tumor microenvironment, which is the underlying mechanism of tissue/disease specificity of circulating miRNAs. Additionally, circulating miRNAs are correlated with the degree of tumor progression and present differently at different stages of cancer, making them play an important role in cancer immunotherapy. That is to say, the presence of certain type of circulating miRNAs was confirmed essentially in the manifestation, development, invasion, and metastasis of cancer, and the abnormal levels of distinct miRNAs could be observed in every process above (Wang et al., 2018). Although the cancer-related biomarkers, which are widely used clinically, are simple and fast, their disadvantages, like poor early diagnosis and prognostic value, limit their role in targeted therapy and the lack of tissue specificity leads to an urgent need to find novel biomarkers. As a result, circulating miRNAs are becoming candidates of emerging non-invasive cellular and molecular biomarkers of cancer.

## Biological Function and Carrier Profiles of Circulating MiRNAs

**Figure 1:**
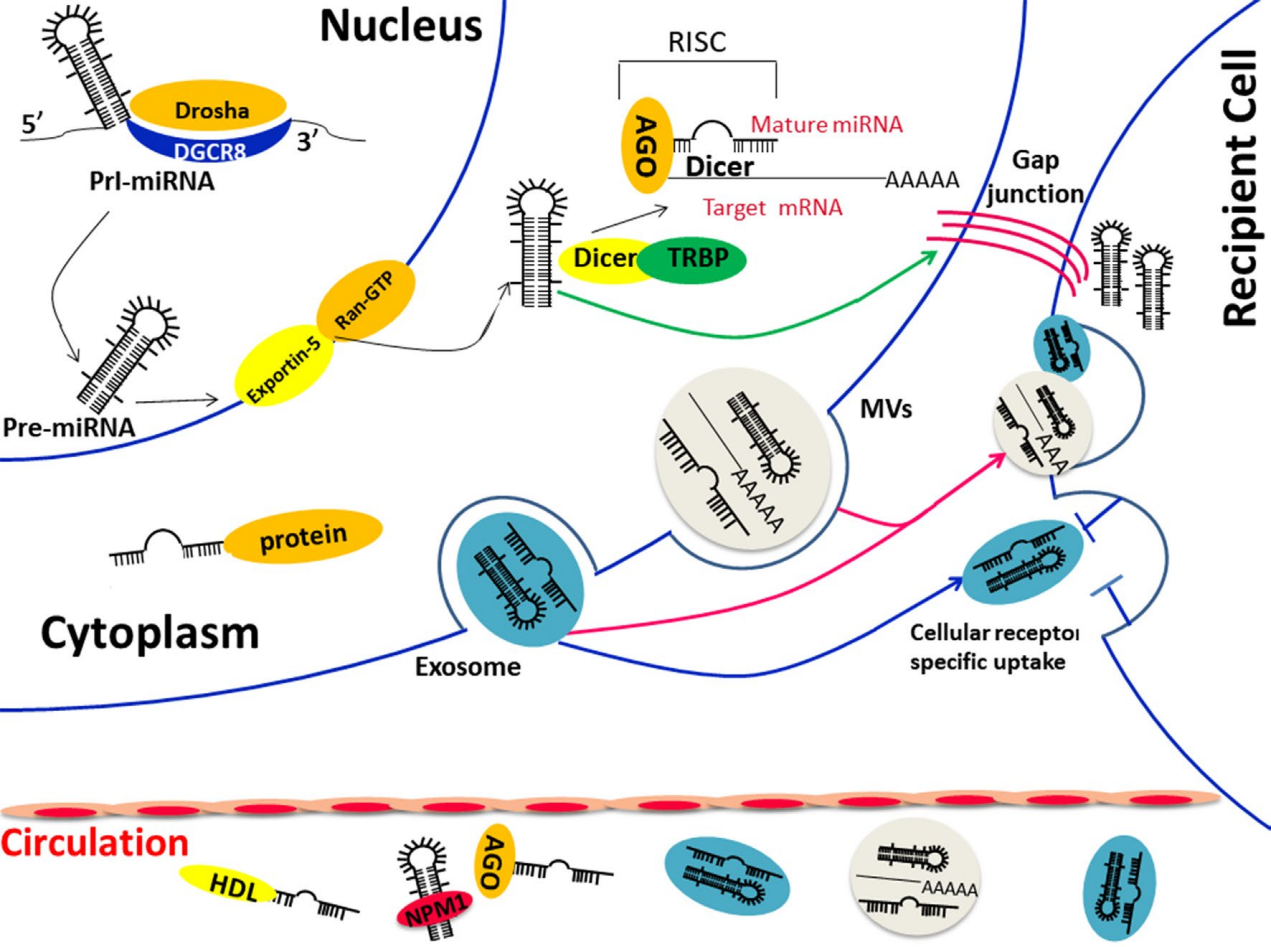
(1) Biological functions and transportation carriers of circulating miRNA; (2) Diverse ways of miRNAs in cell communication: direct fusion and endocytosis of extracellular vesicles (exosomes orMVs) (red arrow) or direct transfer through cell gap junction (green arrow) or the indirect identification of specific surface receptor (blue arrow). Abbreviations: Pre-miRNA, precursor miRNA; Pri-miRNA, primary miRNA; AGO, Argonaute; TRBP, transactivation-responsive RNA-binding protein; DGCR8, DiGeorge Syndrome Critical Region Gene 8; MVBs, multivesicular bodies; NPM1, nucleophosmin 1.

The discovery of circulating miRNA is unanticipated, considering that the activity of RNase in plasma and the underlying mechanism came to be known after years of intensive experiments and discussion. In 2008, Patrick and colleagues confirmed that endogenous miRNAs ranging 18 to 24 nt exist in human plasma by cloning, sequencing, and quantification. Storing at room temperature, suffering freeze-thaw, or extreme variations in pH will not lead to a descending of circulating miRNAs (Duttagupta et al., 2011; Kibel, 2009). However, purified plasma (Arroyo et al., 2011) miRNAs and synthetic miRNAs (Tsui et al., 2002; Kibel, 2009) rapidly degraded when cultured with plasma, suggesting that endogenous miRNAs are resisted to RNase because of various modifications. There are two major populations of circulating miRNAs, vesicle-associated and non–vesicle-associated (**Figure 1**), which reflect the different mechanisms of release. Some tissue-specific miRNAs may release to the circulation in a protein-mediated way and thus presented in protein complex only, such as the liver-specific miR-122 (Chang et al., 2004). However, because releasing vesicles are essential in the process of the maturation and activation of most blood cells, almost all erythrocytes and platelet-related miRNAs are packed into the vesicles in the circulation (Heijnen et al., 1999; Hunter et al., 2010).

Membrane-bound vesicles, such as exosomes (50–90 nm) and microvesicles (1 μm), comprise one type of extracellular miRNA, which can be detected from vesicles isolated and purified from plasma/serum (Valadi et al., 2007; Taylor and Gercel-Taylor, 2008; Hunter et al., 2010). A study done by a team from America found multiple heterogeneous RNAs via Bioanalyzer from exosomes derived from a mast cell line. Large amounts of small RNA were found while the level of ribosomal RNA was low. Then, more than 120 miRNAs were certified in further analysis by microarray (Valadi et al., 2007). There is no doubt that the formation of the miRNA-vesicle package is confirmed by lots of researches. However, recent studies found that the apoptotic body is also one of the forms of the vesicular carrier, but the miRNA it contains may be a random event that differs from the exosome. In a word, all these results indicated the function of MVBs as transporters for extracellular miRNAs.

On the other hand, other studies confirmed that majority of the circulating miRNAs exist in a non–vesicle-associated form, such as the ribonucleoprotein complex. The copies of miRNAs dropped significantly since proteinase K was added to the plasma, which verifies the hypothesis that miRNAs could be degraded by RNase easily when dissociated from a protease-sensitive complex(10), and these proteins, such as Argonaute2 (Ago), GW182, nucleophosmin1 (NPM1), and high-density lipoprotein (HDL), are confirmed as miRNA carriers in a large number of studies. Ago2, the central protein of miRNA-mediated interference, together with GW182, were verified responsible for the protection and transport of extracellular miRNAs (Wang et al., 2010; Arroyo et al., 2011; Yao et al., 2012; Montani and Bianchi, 2016). The miRNA degradation occurred when Ago2 was isolated from the protein complex (Arroyo et al., 2011) and when GW182 was knocked down (Yao et al., 2012). Another protein that takes part in the protection of external miRNAs is the well-known nucleolar RNA-binding protein, nucleophosmin 1 (NPM1), which is involved in the exporting of RNAs and ribosome (Maggi et al., 2008). Synthetic miR-122 was not degraded by RNase when incubated with NPM1, and the NPM1 was confirmed not only participated in the packaging of miRNAs but also in the process of miRNA exporting (Wang et al., 2010). High-density lipoprotein is widely known as a mediation of excess cellular cholesterol. However, its function is far more complex especially when it involves the transportation and post-transcription effect in the recipient cells of miRNAs (Rothblat and Phillips, 2010; Vickers et al., 2014). As a result, HDL has been used as the delivery of siRNAs in animal models (Kim et al., 2007).

In conclusion, it is just these miRNA transporters that protect circulating miRNAs from RNase in various body fluids. It may be associated with the cell type and tissue specificity, which carrier miRNAs “select” and the carrier of one specific miRNA may be not unique (Arroyo et al., 2011; Li et al., 2012).

## Circulating MiRNAs in Cell Communication

Traditional cell–cell communication means gap junctions or cell signal transduction, such as neurotransmitter, hormone, and cytokines, whereas the extracellular miRNA-dependent cell-cell communication is proven to be induced by membrane-derived vesicles recently (Xu et al., 2013). Exosomes were demonstrated to be related to the immune function many years ago, and researchers were surprised to confirm that both mRNA and miRNAs could be packaged to the “magical” vesicles and are exported or released from cells in response to biological stimuli (Cortez et al., 2011). The lipid bilayer is the coat of exosome that reflects the information from the original cell, and the proteins on the surface are important in cell signal pathway (Zhao et al., 2015). Respiratory chain inhibitor rotenone was able to reduce the level of extracellular miRNAs (Wang et al., 2010), and the mRNA and miRNA profiles of exosomes are different from those of the parent cells (Valadi et al., 2007), suggesting that the secretion of exosomes is an ATP-dependent and active-selecting process. On the other hand, the difference between the spectra of intracellular and extracellular miRNAs under the condition of no cytolysis indicated that the cellular control mechanism was involved in the release of extracellular miRNAs(20). In *in vitro* experiments, many miRNAs were overexpressed in the culture medium after the intervention of serum starvation and proved to be related to the cell cycle arrest, apoptosis, and cell death, suggesting that the biological function of miRNAs may extend outside of the cell and mediate cell–cell communication (Wang et al., 2010). A few selected miRNAs showed a trend of translating from intracellular to extracellular during the first 2 h of serum elimination, which suggested that there is a process of prepackage and storage of miRNAs (Wang et al., 2010). Those encapsulated miRNAs are able to reach the remote area and affect recipient cells, especially various immune cells in the tumor microenvironment, which is important for tumorigenesis (Gong et al., 2012; Lässer, 2012). miRNAs could participate in a directional transfer from T cells to antigen-presenting cells using exosomes as a carrier. Not surprisingly, miRNAs in exosomes are induced by immune cells different from their parent cells, indicating that miRNAs were changed in the process of intercellular communication during immune interactions (Mittelbrunn et al., 2011). Other immune cells, such as dendritic cells (DCs), were proven to transfer signals to neighboring DCs via exosome shuttle miRNAs, and the packed miRNAs were different according to the maturation of the DCs (Montecalvo et al., 2012). That is to say, exosomal miRNAs are one of the complex strategies of immune cell communication. Although the exact underlying mechanism of exosomal miRNAs in cell communication is still unclear now, we have obtained evidence of several hypotheses (**Figure 1**): 1) direct fusion of vesicles and receptor cell membranes (Mulcahy et al., 2014), 2) active endocytosis or phagocytosis by receptor cells, 3) identification of proteins on the surface of exosomes and receptors of recipient cells (Munich et al., 2012; Corrado et al., 2014), 4) cell gap junction-mediated transfer (Lim et al., 2011; Aucher et al., 2013). The communication between circulating miRNAs and target cells will lead to a series of effects on both physiological and pathological conditions (Kosaka et al., 2010; Pegtel and Kieff, 2010) and the packed miRNAs make an exchange of genetic material additionally (Valadi et al., 2007). Some miRNAs exist in exosomes not derived from their parental cell. These have confirmed that the gene transfer is mediated by exosomes (Valadi et al., 2007; Zhang et al., 2015a). The extracellular miRNAs not only affect the surrounding cells but also the distant tissues, thereby leading to the progression of diseases (Mathivanan et al., 2012).

Adipose tissue contains a different type of circulating exosomal miRNA that can regulate distant cells as a form of adipokine, which is confirmed by the experiment that miRNAs expressed in the brown adipose in one mouse could regulate its target liver reporter in other mouse (Thomou et al., 2017). Additionally, accumulating evidence has shown that circulating miRNAs participate in the invasion and metastasis of cancer via cell communication with recipient cells (Mei et al., 2011). Secreted miRNAs from metastatic cells were transported to endothelial cells and promoted angiogenesis, which are regulated by neutral sphingomyelinase 2 (Kosaka et al., 2013a). All of these studies indicate that (Lee et al., 1993) sending information via extracellular miRNA is another way of intercellular communication (Wightman et al., 1993), miRNA-dependent cell–cell communication is the best explanation for the existence of circulating miRNA, and (Bartel, 2004) circulating miRNAs secreted by cancer cells can trigger tumorigenesis in the recipient cells.

## Circulating MiRNAs as Promising Cancer Biomarkers

miRNAs are a family of endogenous 19-22nt noncoding RNAs involved in posttranscriptional regulation and participate in various physiological and pathological processes by inhibition of translation or by mRNA cleavage. More than 50% of protein-coding genes are assumed to be the target of miRNAs (Krol et al., 2010). The miRNA expression is frequently dysregulated in cancer, forming tissue-specific expression patterns. In addition to their intracellular biology functions, numerous studies have documented that the dysregulation of extracellular miRNAs is associated with the origin, progression, therapeutic response, and patient survival of the disease since the presence of circulating miRNAs in serum was first described (Wang et al., 2018; Zhang et al., 2018). Mouse prostate cancer xenograft model was used to identify whether the abnormal expressed serum miRNA was tumor-derived in *in vivo* experiment. Results showed that the levels of miR-620 and miR-629 in the serum were different and can distinguish cancer-bearing mice from controls (Mitchell et al., 2008). There was a surprisingly distinct miRNA expression between endothelial cells cultured with cancer cell lines with control, which means the upregulated miRNAs were induced by tumor cells (Zhuang et al., 2013). At the same time, this study confirmed the hypothesis that stimulated miRNAs were packed into microvesicles and delivered to endothelial cells in follow-up experiments (Skog et al., 2008; Zhuang et al., 2013). On the other hand, the phenomenon that tumor-suppressing miRNAs were amplified, whereas oncogenic miRNAs were reduced, reflected the fact that circulating miRNAs were not the primary products of cancer cells, but the results of global immune response and play an important role in cancer defense and cancer therapy (Aucher et al., 2013). However, another phenomenon is that cancer cells transfer the intercellular tumor-suppressive miRNAs to the extracellular environment, modify tumor microenvironment, and support cancer progression. In other words, extracellular miRNAs can act as both oncomir and suppressor by different stimuli. These studies provided the evidence that miRNAs may derive from tumor cells in response to specific signals, enter the circulation in a stable form as cancer-related molecules, and contribute to early diagnosis, prognosis, and individualizing therapeutic strategies (**Table 1** (Gonzales et al., 2011; Silva et al., 2011; Zhou et al., 2011; Bryant et al., 2012; Sun et al., 2012; Valladares-Ayerbes et al., 2012; Kawaguchi et al., 2013; Ng et al., 2013; Nguyen et al., 2013; Takeshita et al., 2013; Tanaka et al., 2013; Wang et al., 2013; Zeng et al., 2013; Enache et al., 2014; Geng et al., 2014; Ogata-Kawata et al., 2014; Zanutto et al., 2014; Zhang et al., 2014; Chen et al., 2014a; Wang et al., 2014; Antolin et al., 2015; Stuckrath et al., 2015; Zhang et al., 2015b; Zhang et al., 2016; Mirzaei H. et al., 2016; Mirzaei H. R. et al., 2016; Zhang et al., 2017; Zhao et al., 2017; Bahrami et al., 2018; Jamali et al., 2018; Shekari et al., 2018; Wang et al., 2018).

**Table 1:** Values of circulating miRNAs in different cancers.

Cancers	Expression profile and treatment value	Diagnostic value					Prognostic value			Diagnostic and prognostic value		RE
Gastric cancer	upregulated	miR-421 miR-20a miR-103 miR-181c	miR-378 miR-221 miR-744 miR-192	miR-199a-3p miR-486-5p miR-199a-3p miR-423-5p	miR-107 miR-194 miR-17 miR-1	miR-34a miR-27a miR-185 miR-210	miR-148a miR-146a miR-218 miR-18a	miR-214 miR-301a miR-223 miR-16	miR-100 miR-451 miR-106a miR-222	miR-21 miR-25 miR-200c		52-56
	downregulated	miR-195-5p miR-17-5p	let-7a	miR-106b	miR-375	miR-320a	miR-218 miR-203	miR-93 miR-92b	miR-19b-3p miR-16-5p	miR-196a miR-122		
Esophageal cancer	upregulated	miR-223-3p miR-192-5p miR-28-3p	miR-223 miR-22 miR-21	miR-127-3p miR-296-5p miR-20b-5p	miR-10a miR-100	miR-148b miR-133a	miR-367	miR-200c		miR-1246 miR-146a		57-60
	downregulated	miR-100-5p miR-375					miRNA-718					
Pancreatic cancer	upregulated	miR-378* miR-409-3p miR-1290 miR-26a miR-18a					miR-146b-3p miR-200a miR-200c miR-210 miR-221 miR-21 miR-194			miR-141 miR-375		61-63
	downregulated	let-7b-5p let-7c-5p					miR-409-3p					
Breast cancer	upregulated	miR-195 miR-376c miR-409-3p miR-148b miR-299-5p		miR-145 miR-191 miR-382 miR-215	miR-133a miR-133b miR-92a miR-192	miR-1 miR-411 miR-195 miR-202	miR-122 miR-141			miR-21 miR-34a miR-210 miR-10b	miR-375 miR-125b miR-801 miR-155	64-68
	downregulated	miR-181a-5p miR-34 miR-92a		miR-139-5p miR-143 miR-133a	miR-30a let-7a	miR-145 miR-365	miR-375 miR-30a miR-205	miR-342-5p miR-200c	miR-497 let-7b	miR-768-3p		
	Therapeutic target	miR-155 (upregulated) miR-214 (downregulated)										
HCC	upregulated	miR-122 miR-801 miR-885-5p		miR-192 miR-223 miR-130b	miR-18 miR-15b	miR-26a miR-27a	miR-221 miR-1					69-71
	downregulated	miR-16 miR-199a miR-21										
Prostate cancer	upregulated	miR-378* miR-409-3p miR-1290 miR-26a miR-18a					miR-146b-3p miR-210 miR-21 miR-221 miR-19 miR-200a miR-200c			miR-141 miR-375		72-74
	downregulated	let-7b-5p let-7c-5p								miR-409-3p		
NSCLC	upregulated	miR-20a-5p, miR-141-3p, miR-145-5p,		miR-155-5p, miR-223-3pmiR-126-3p	miR-210-3p miR-16-5p	miR-182-5p, miR-183-5p,	miR-320b miR-23b-3p miR-10b-3p		miR-195-5p miR-4257-3p miR-222-3p	miR-21-5p 39 40 41 30 31		11 75-78
	downregulated	miR-198 miR-361-3p miR-625					let-7f miR-30e-3p							
	Therapeutic target	miR-181-5p miR-361-5p miR-205-5p miR-10b miR-30a-3p miR-30e-3p miR-15b miR-9-5p										
Colon cancer	upregulated	miR-27a-3p, miR-142-5p miR-409-3p miR-223		miR-92a miR-601 miR-760 miR-18a	miR-1229 miR-1246 miR-150 let-7a	miR-7 miR-93 miR-29a miR-23a	miR-29c miR-200c miR-20a miR-130		miR-145 miR-216 miR-372 miR-378	miR-23a-3p miR-376c-3p mi-221 miR-141 miR-21		79-82
	downregulated	miR-125a-3p miR-34a		miR-181b miR-92a	miR-601 miR-760	miR-203 miR-31	miR-4772-3p					

### Circulating MiRNAs as Biomarkers for Early Diagnosis

The diagnosis of cancer currently suffers from low sensitivity, because many tumors cannot be found at the early stage and delay the treatment until it is too late. MiRNA expression is frequently dysregulated in cancer, forming a particular expression profile and, thus, benefits the early detection of cancer. MiRNAs associated with tumor growth are highly expressed, whereas suppressors are expressed lower. Therefore, these tissue-specific miRNAs are becoming emerging candidates in cancer diagnosis. Selected plasma/serum circulating miRNAs could be used to discriminate various cancer patients from healthy individuals such as breast (Antolin et al., 2015), colorectal (Zanutto et al., 2014), gastric (Zhang et al., 2015b), lung (Zhao et al., 2017), pancreatic (Kawaguchi et al., 2013), and hepatocellular (Mirzaei H. R. et al., 2016) cancers, making them tools for earlier diagnosis. In addition, differential concentrations of miRNAs were detected among different cancer subtypes and differentiation grades in breast cancer; aberrant levels of miRNAs were associated with the HER2 and estrogen receptor status as well, indicating the diagnostic and therapy-selecting potential of circulating miRNAs (Stuckrath et al., 2015). The expression of miR-21 was associated with the clinical stage and molecular subgroup of diffuse large B-cell lymphoma (DLBCL), which means that patients in early stage have a higher concentration of serum miR-21 than those in stage III and IV and patients with different subgroup have an obvious differentiation (Chen et al., 2014b). On the other hand, miRNAs show a remarkable relationship with tumor derivation, which is important in the identification of metastatic tumors with unknown primary origin. A microarray of 48 selected miRNAs could trace and classify 90% primary tumor in metastatic samples (Rosenfeld et al., 2008). The combination of miR-145 and miR-451 could discriminate breast cancer from healthy individuals as well as other types of cancers, including liver cancer, lung cancer, and colorectal cancer, which validated the function of circulating miRNAs in cancer classification (Ng et al., 2013). Furthermore, not only cancers could be distinguished from normal ones but also those who suffered from chronic inflammation were singled out by a distinct miRNA expression pattern. Recent studies suggest that circulating miRNAs are involved in the regulation of inflammation, influence the genetic/epigenetic profile, and capable of predicting the unhealthy incidents (Olivieri et al., 2016). For example, a selected miRNA expression panel could differentiate pancreatic cancer from chronic pancreatitis with relatively high accuracy (Bloomston et al., 2007), whereas it can be used as biomarkers in the identification of hepatitis B virus (HBV) infection and HBV-positive hepatocellular cancer (Li et al., 2010). However, what needs to be pointed out is that the same miRNA can act as either oncogene or suppressor gene, depending on different cancer types (Cortez et al., 2011). MiR-125b could suppress cell proliferation and induce cell cycle arrest in ovarian, thyroid, and oral cancers (Visone et al., 2007; Nam et al., 2008), whereas it functioned oppositely in prostate cancer (Le et al., 2009). As a result, it is important to find out the corresponding abnormally expressed miRNAs in every type of cancers.

On the other hand, the phenomenon illustrated that some of the miRNAs are aberrantly expressed in tumors with an obvious familial aggregation tendency that can be applied to genetic diagnostics. MiR-15 and miR-16 are down-regulated in most of the B-cell chronic lymphocytic leukemia (B-CLL) patients due to the 30-kb region of loss in chromosome 13q14, which is the most frequently deleted genomic region of B-CLL (Calin et al., 2002). Similarly, acute myeloid leukemia patients with chromosomal translocations were proved together with a low level of miR-223 (Fazi et al., 2007). Even single nucleotide polymorphisms (SNPs) in miRNA genes may affect the biogenesis of miRNAs and thus increase the risk of cancer. SNP (rs417309), located in the 3′-UTR of DGCR8, was consistently associated with the possibility of suffering from breast cancer by the mechanism of interrupting the binding of miRNA, whereas an SNP in let-7 complementary sites could increase the risk of non-small cell lung cancer (Chin et al., 2008) as well. These results are also consistent with the studies that genes of miRNAs are most often located at fragile sites and genomic regions associated with cancers (Calin et al., 2004).

### Circulating MiRNAs in the Prediction of Prognosis

A large number of studies have suggested the prognostic and predictive values of cancer-related circulating miRNAs as they participate in the regulation of the development of cancer. In the progression of cancer into a more invasive phenotype, miRNAs change as molecular labels of tumor cells, and the changes can be observed from tumorigenesis throughout the following progression. Therefore, circulating miRNAs are one of the most reliable candidates in disease monitoring. Circulating miR-142-3p correlated with a high risk of recurrence in lung adenocarcinoma patients of early stage (Kaduthanam et al., 2013). The levels of serum miR-155 could reflect the effect of surgery and chemotherapy in breast cancer, whereas the conventional biomarkers, such as carcinoembryonic antigen (CEA) and tissue polypeptide-specific antigen (TPS), were not that sensitive (Sun et al., 2012). Altered circulating miRNAs have also been proven to be bound up with the metastasis of cancer, and miR-141 achieved positive results in a test in prostate cancer patients in terms of identification of micro-metastasis (Gonzales et al., 2011). Decreasing levels of cir-miRNA-126 were related to treatment benefit in metastatic colorectal cancer, as it was proven to be associated with angiogenesis by way of paracrine (Hansen et al., 2015). Similarly, higher levels of circulating miR-122 have a positive correlation with the metastatic recurrence in stage II–III breast cancer patients (Wu et al., 2012). MiR-375 and miR-200b in the serum were significantly upregulated in patients with metastatic prostate cancer compared with patients with localized cancer (Bryant et al., 2012). Some of the other miRNAs that influence the epithelial phenotype of cancer cells were found elevated in the blood of gastric patients and induce invasion and migration (Valladares-Ayerbes et al., 2012). Additionally, circulating miR-214 and miR-373 were related to lymph node metastasis as well (Chen et al., 2013). Responsive miRNAs were observed valuable in therapy monitoring in head and neck squamous cell carcinoma (Summerer et al., 2013).

On the other hand, the changes of circulating miRNAs during chemotherapy and radiotherapy of cancer are well appreciated in many studies. Non-small-cell lung cancer (NSCLC) patients with clinical stage Ib to IIIa often need comprehensive treatment including operation and chemotherapy; a prediction for drug and chemotherapy sensitivity in advance can reduce the unnecessary toxic chemotherapy. A selected serum miRNA panel may serve as a predictor for the purpose of the above and found to be associated with the overall survival of NSCLC (Hu et al., 2010). Serum miR-125 and miR-22 led to the poor response to cisplatin-based and pemetrexed-based chemotherapy separately in NSCLC patients (Cui et al., 2013; Franchina et al., 2014). Serum miR-21 was associated with the relapse-free survival in DLBCL (Lawrie et al., 2008). MiR-150 was sensitive to acute radiation exposure and thus useful for the evaluation of treatment and toxic dose, which is essential for clinical radiation therapy (Jacob et al., 2013). All these studies suggested that circulating miRNAs are promising invasive biomarkers and are considered to be valuable in tumor classification, treatment strategy selection, cancer prognostication, and monitoring.

### Circulating MiRNA-Based Cancer Therapy

Currently, more and more studies focus on the biological behavior of circulating miRNAs. It was found that not only mRNAs and proteins that are packed into the MVs or exosomes, but also miRNAs are proved to be existing in the MVs and exosomes abundantly. These exosomes secreted by donor cells containing packed miRNAs could be taken by recipient cells, both in the surrounding and remote area, which is the theoretical basis of miRNA-based targeted cancer therapy via vesicles. However, the delivery method of miRNA is an essential problem to resolve for RNAi therapy. Compared with carriers of targeted therapy, such as viruses, lipid, and polymeric nanoparticles, microvesicles serve as a natural carrier and could avoid attack from the immune system (Cheng, 2015), which is quite important in the persistence of drug intervention. Therefore, MVs and exosomes are used for the delivery of therapeutic RNAi as a more effective strategy in cancer therapy (Kosaka et al., 2013b). In other words, the transfer of synthesis tumor-suppressive miRNAs or antisense of tumor oncogenesis miRNAs into target tumor cells through MVs or exosomes deserves continuing concern. MiR-150 is an immune-related miRNA and participates in the secreting of vascular endothelial growth factor via the regulation of tumor-associated macrophages and plays positive role in tumor growth. Experts transfer antisense miR-150 to MVs and inject “modified” MVs to mice via tail. Results showed that MVs could deliver the antagonucleotide for onco-mirna into tumor efficiently, and thus prevent tumorigenesis (Liu et al., 2013). Using GE11, a kind of peptide that can bind to the epidermal growth factor receptor, miR-let-7a reached the breast cancer tissue of xenograft mice model specifically and inhibited tumor development *in vivo* successfully (Ohno et al., 2013). MVs derived from human adult liver stem cells containing several anti-miRNAs could inhibit the growth of the tumor (Valentina et al., 2012). Furthermore, anti-mRNAs were also effective in drug resistance. For example, miR-9 was associated with the drug efflux transporter and was found upregulated in temozolomide-resistant glioblastoma multiforme (GBM) cells, whereas mesenchymal stem cells (MSCs)-derived exosomes complete the key biological processes of transferring anti-miR-9 from MSCs to GBM cells and reverse the chemoresistance finally (Munoz et al., 2013). Taken together, tumor-suppressive miRNAs could be delivered to target cancer cells *in vivo* and can be promising small RNAs for cancer therapy. However, the decomposition by the reticuloendothelial system and the inappropriate immune responses are the major issues of miRNA-relevant cancer therapy before their application.

## Challenges in Using Circulating MiRNAs as Cancer Biomarkers

As we discussed above, circulating miRNAs are becoming potential non-invasive biomarkers for the prediction, prognosis, and therapeutic targets for cancers. Despite their many advantages, there are still challenges to overcome before clinical application.

### Technical Challenges

The fundamental technical constraint to solve is the isolation and purification of samples, as the integrity and purity of RNA are the basic of detection and quantification. Unlike intercellular miRNAs, circulating miRNAs are interference by other components in serum easily and need to be cautious when centrifuged from serum (Cheng et al., 2013). It is necessary to add a step for purification as cell-free miRNAs are modified with exosomes, microvesicles, AGO2/NPM1, and HDL (Lee et al., 1993). Besides, the storage time and conditions also impact the composition of miRNAs; the level of several miRNAs, including miR16, is changed after 24 to 72 h storage either in the situation of 4°C or −20°C (McDonald et al., 2011) and brings another challenge in sample processing. That is to say, different experimental setups and processes all lead to the bias in the final output of miRNAs (Cortez et al., 2011; Schwarzenbach et al., 2014; Lee et al., 2016). Validated and optimized experimental protocols are needed urgently.

Second, the source of samples is also one of the most critical aspects of the ultimate results of circulating miRNAs (Wang et al., 2012). The expression of miRNAs is different between the samples extracted from the serum and plasma even in the same individual. The total RNA concentration is higher in the serum than in the plasma, which may be due to the RNA released from blood cells and platelets (Wang et al., 2012). However, analysis results from studies did not distinguish sample types (serum/plasma) when grouping.

Third, it is still hard to measure circulating miRNAs accurately because of its low concentration and existing form. The major quantifiability detection of circulating miRNAs are qPCR, microarray, and next-generation sequencing (NGS). Quantitative PCR is limited by low throughputs. It is now widely used for the verification of sequencing data. Microarray is influenced by the short length and similar sequence among clusters and families of mature miRNAs. Its requirement of pre-amplification step has a risk of alliterating the actual concentration of circulating miRNAs (Chen et al., 2009). NSG can meet the low concentration of circulating miRNAs due to its low input request and become a preferred method because of its lower cost and higher throughputs. Therefore, it is indispensable to unify the measurement methods and eliminate the deviation.

Additional obstacle lies in the normalization of data, especially the selection of internal control. U6 is widely used for intracellular miRNAs, but it is restricted due to its low expression in body fluids (Singh et al., 2016). Mir-16 was mentioned in many studies, and the inconsistent results also appear in multiple myeloma (Wang and Chen, 2014). Other internal controls, such as UNR6B (Xiang et al., 2014) and miR39 (Wulfken et al., 2011), are not trustworthy yet. The additive artificial non-human miRNAs external control like cel-miR-39 and cel-miR-54 (Zhong et al., 2018) could be a choice to solve this problem. However, it is hard to balance the amount among different samples. On the other hand, because of the expression of extracellular miRNAs in healthy individuals and the variability in acute/chronic inflammation/injury, the identification of a range of negative and cancer-specific “diagnostic” miRNAs is necessary before miRNAs can become clinical test indicators (Zhao et al., 2010; Xiang et al., 2014).

### Cognitive Challenges

In addition to the technical challenges, the unclear understanding of the function and biology characteristics of circulating miRNAs, such as the secretion and transportation mechanisms, the cell to cell communication, the complicated network between miRNAs and coding gene, and the effects in upstream/downstream pathways are great barriers before clinical transformation (Wang and Chen, 2014). In the past few years, many researchers have devoted to the precise mechanisms of the secretion and uptake of miRNAs. However, it is still not clear if the package of miRNAs is random or specific; the release of cell-free miRNAs is passive or active. Besides, molecules and signals that are involved in the regulation of miRNAs, the precise role of circulating miRNAs in oncogenesis, the great heterogeneity of miRNAs in each type of cancer, different tumor stages, treatment response, and survival are all tasks that require more investigations.

At the same time, there are still challenges in the usage of circulating miRNAs in targeted therapy. Packaged artificial and modified miRNAs in exosomes could increase the stability of miRNAs *in vivo* (Cortez et al., 2011). However, the restricted tissue specificity and permeability is a big problem. Ligand, antibody, and nanoparticles that carried miRNAs are designed nowadays with improved specificity and decreased immunotoxicity (Chen et al., 2015). Nevertheless, a large number of preclinical studies in animals should be considered to verify their effectiveness.

## Conclusions and Perspectives

Since the first discovery of circulating miRNAs, there is a large amount of studies focused on their biological functions and the potential of biomarkers in oncology. As described here, miRNAs in the circulation change as molecular labels of tumor cells throughout the tumorigenesis and development of cancer. Such detectable changes make circulating miRNAs promising non-invasive biomarkers for early cancer diagnosis and predictor of prognosis and cancer treatment. However, several issues including technical and non-technical constraint need to be solved urgently. The further understanding of existing form in circulation and biological function, the deeper exploration of the underlying mechanism of release, transport, and uptake, and the special status in cell communication are all essential before the breakthrough in the application of circulating miRNA-based cancer therapy.

## Author Contributions

RC and XZ conceived the idea. MC wrote the manuscript. HW and XY revised the manuscript. DZ and YX edited the manuscript.

## Funding

This work was supported by Program for JLU Science and Technology Innovative Research Team.

## Conflict of Interest Statement

The authors declare that the research was conducted in the absence of any commercial or financial relationships that could be construed as a potential conflict of interest.
